# In vitro differentiation and phenotypic profiling of Langerhans cell subsets derived from human peripheral blood monocytes

**DOI:** 10.1007/s12026-026-09825-8

**Published:** 2026-09-07

**Authors:** Sylwia Hasterok, Klas Berggren, Claes af Klinteberg, Håkan Eriksson, Lars Ohlsson, Anna Gustafsson

**Affiliations:** 1 Department of Biomedical Science, Faculty of Health and Society, Malmö University, Malmö, Sweden; 2 Biofilms – Research Center for Biointerfaces, Malmö University, Malmö, Sweden; 3 Lumito AB, Lund, Sweden

**Keywords:** Langerhans cells, Subset heterogeneity, Human peripheral monocytes, In vitro method

## Abstract

Langerhans cells (LCs) are primary immune sentinels at barrier surfaces, acting as the first line of defence against foreign pathogens. However, their low abundance within the human epidermis severely limits their availability for functional and mechanistic studies. To address this challenge, we sought to provide a versatile experimental framework for generating, identifying, and characterizing distinct subsets of LCs derived from in vitro differentiation of human peripheral monocytes. LCs’ heterogeneity has long been considered, with recent studies classifying them into four subsets: effector LCs (LC1) and regulatory LCs (LC2) present at steady state, along with activated LCs (aLC) and migratory LCs (migLC) found during skin inflammation. In this study, LC1, LC2, and aLC/migLC subsets were identified by flow cytometric profiling of CD207, CD1a, CD83, and CD197. LC phenotype was further confirmed by examining HLA-DR, CD14, CD209, CD1b, and CD1c expression. Additionally, the phagocytic capacity of LCs relative to distinct macrophage polarizations was evaluated. From the in vitro-generated LCs, the subsets LC1, LC2, and a joint aLC/migLC cluster were identified. LC1 constituted the predominant population, appearing at higher proportions than both LC2 and the aLC/migLC cluster. The proportions of LC2 and aLC/migLC remained relatively consistent across donors, whereas LC1 abundance showed greater variation, suggesting a higher donor-dependent heterogeneity. Overall, LCs exhibited the lowest phagocytic capacity compared with the different macrophage polarizations. Our study defines LC subsets that can be generated from peripheral monocytes in vitro and provides a framework for deeper exploration of human LC biology, subset heterogeneity, and function in both normal and pathological skin.

**Supplementary Information:**

The online version contains supplementary material available at https://doi.org/10.1007/s12026-026-09825-8.

## Introduction

Langerhans cells (LCs) are tissue-resident immune cells that play a critical role in immune surveillance at the interface between the internal and external environments, acting as a first line of defence against foreign pathogens [1, 2]. Within the skin, LCs are located at the *stratum spinosum* of the epidermis, where they constitute 3–5% of all nucleated cells. There, through their dendritic extensions, LCs engage in close crosstalk with keratinocytes, the primary building blocks of the epidermis, and form an intricate cellular immune network [2, 3].

The skin barrier is a site of persistent exposure to mechanical, chemical, radiative, and biological factors; therefore, LCs modulate their activity to maintain immune tolerance with pro-inflammatory responses in the epidermis [2]. This is relevant because, in quiescent human skin, LCs are the only hematopoietic cells residing in the epidermis, positioning them as the primary immune sentinels at the barrier surface and key regulators of local immune homeostasis [4].

Severe local inflammation promotes LC activation by enhancing antigen processing and upregulating major histocompatibility complex II (MHC II) and C-C chemokine receptor type 7 (CCR7; CD197). This results in extensive LC migration from the epidermis to draining lymph nodes, where they take on the role of antigen-presenting cells (APCs) and initiate adaptive immune responses [5, 6]. The one-way mass exodus of LCs from the epidermis upon substantial inflammation is not without consequence, as it results in a marked depletion of the skin-resident LC pool, thereby compromising immune surveillance at the epidermal barrier [6]. Therefore, to restore the continuity of the immune defense and re-establish homeostasis, repopulation of the epidermal LC niche is supported by a resident myeloid LC population, persistent within the epidermis, through slow, in situ proliferation, and by circulating monocytes differentiating into LCs [7].

Under non-inflammatory conditions, epidermal LCs promote the activation and proliferation of skin resident regulatory T cells (Tregs). In parallel, the steady state LC population is maintained via slow in situ proliferation of mature embryo-derived LCs (eLCs), enabling gradual self-renewal. Simultaneously, a small number of LCs migrate to draining lymph nodes, where they present self-antigens to T cells, contributing to the establishment of immune tolerance under homeostatic conditions [4, 8].

Intra-population heterogeneity has long been considered as a potential explanation for the diverse functional roles of LCs in maintaining skin homeostasis [3, 9], with evidence supporting both immunogenic and tolerogenic activities [10, 11]. In line with this notion, recent studies have confirmed that LCs do not constitute a uniform cell population in the human skin [12, 13]. As a result, four distinct LC subsets have been defined, including: effector LCs (LC1) and regulatory LCs (LC2) present under steady-state conditions, as well as activated LCs (aLC) and migratory LCs (migLC) found during inflammation [12, 14]. Each of these LC subsets is characterized by distinct transcriptomic, phenotypic, and functional profiles [12].

The classification of LCs and their developmental origin has long been a subject of debate, as they exhibit a unique combination of features characteristic of both macrophages and dendritic cells (DCs) [2]. Subsequently, much of the current understanding of LC biology comes from research conducted in murine models [14]. However, while mice exhibit roughly 85% genomic similarity to humans, functional and phenotypic differences in the skin immune system exist, limiting the translational value of experimental results to humans [15–17]. Moreover, animal testing continues to raise significant ethical concerns about the welfare and rights of animal subjects [18]. Considering the 2025 release of new guidelines advocating stricter adherence to the 3R principles (Replacement, Reduction, and Refinement), the development and adoption of human-relevant experimental models that can replace or reduce animal testing are expected to increase [19, 20].

The aim of this study was to establish and validate a versatile experimental framework that enables the generation, identification, and detailed characterization of distinct LC subsets derived from human peripheral blood monocytes in vitro.

## Materials and methods

### Langerhans cell differentiation

#### Day 0 – classical monocyte isolation from PBMCs

Leukocyte concentrates obtained from healthy volunteer donors were purchased from a local blood donation center (Skånes universitetssjukhus, SUS, Lund, Sweden). Peripheral blood mononuclear cells (PBMCs) were then isolated by density gradient centrifugation using Ficoll-Paque™ (GE Healthcare Life Sciences, Danderyd, Sweden). Next, highly purified classical monocytes were isolated using the Classical Monocytes MACS Isolation Kit (Miltenyi Biotec, Bergisch Gladbach, Germany), following the manufacturer’s protocol. After purification, classical monocytes were diluted to the concentration of 1 × 10^6^ cells/mL in RPMI 1640 medium (1X) with GlutaMAX^TM^-I (Gibco, Thermo Fisher Scientific, Waltham, MA, USA), supplemented with (v/v) 10% fetal calf serum (heat inactivated FCS, Gibco, Thermo Fisher Scientific) and 50 µg/mL gentamycin sulfate (Corning, Corning, NY, USA). This culture medium is referred to as R10. Next, the isolated monocytes were seeded onto 12-well plates (1 mL/well; VWR International, LLC, PA, USA), 96-well plates (0.1 mL/well; VWR International), BD culture chamber (0.3 mL/well; BD Falcon Culture Slides, uncoated, BD Biosciences, San Jose, CA, USA), and incubated for 2 h at 37 °C to ensure cell adherence. This was followed by removal of the culture medium from the 12-well and 96-well plates and the BD culture chamber, and its replacement with the appropriate volume of R10-IL-4 + medium, containing 100 ng/mL GM-CSF (Miltenyi Biotec), 10 ng/mL TGF-β (Miltenyi Biotec), 10 ng/mL TNF-α (Miltenyi Biotec), and 10 ng/mL IL-4 (Miltenyi Biotec). The cells were then incubated at 37 °C in a humidified atmosphere with 5% CO_2_ for 2 days.

#### Day 2 – complete IL-4 removal from the medium and cell retention

The 12-well and 96-well plates and the BD culture chamber were gently swayed to detach loosely attached cells. The medium containing non-adherent cells was carefully collected from all wells and the chamber, pooled into a 50 mL tube (Corning), and centrifuged at 300 × g for 5 min. Meanwhile, the culture plates and the chamber with adherent cells were replenished with 0.5 mL, 0.1 mL, and 0.15 mL of R10-IL-4-free medium containing 100 ng/mL GM-CSF, 10 ng/mL TGF-β, and 10 ng/mL TNF-α. After centrifugation, the supernatant from the pooled non-adherent cells was discarded and replaced with half the original volume of R10-IL-4-free medium containing 100 ng/mL GM-CSF, 10 ng/mL TGF-β, and 10 ng/mL TNF-α. The cells were then resuspended, thoroughly mixed, and redistributed into each well of the culture chamber (0.15 mL/well), 96-well (0.1 mL/well) and 12-well plates (0.5 mL/well) containing adherent cells and incubated for 2 days in a humidified atmosphere (37 °C, 5% CO_2_).

#### Day 4 – partial replenishment of culture medium

The 12-well and 96-well plates and the culture chamber were carefully tilted to remove half of the original volume of the R10-IL-4-free medium (0.5 mL/well in a 12-well plate, 0.15 mL/well in a BD culture chamber, and 0.1 mL/well in a 96-well plate). The extracted volume was then replaced with an equal amount of fresh R10-IL-4-free medium containing 100 ng/mL GM-CSF, 10 ng/mL TGF-β, and 10 ng/mL TNF-α. The cells were then incubated at 37 °C in a humidified atmosphere with 5% CO_2_ for 2 days.

#### Day 6 – LC phenotyping using CD markers

**Fig. 1:**
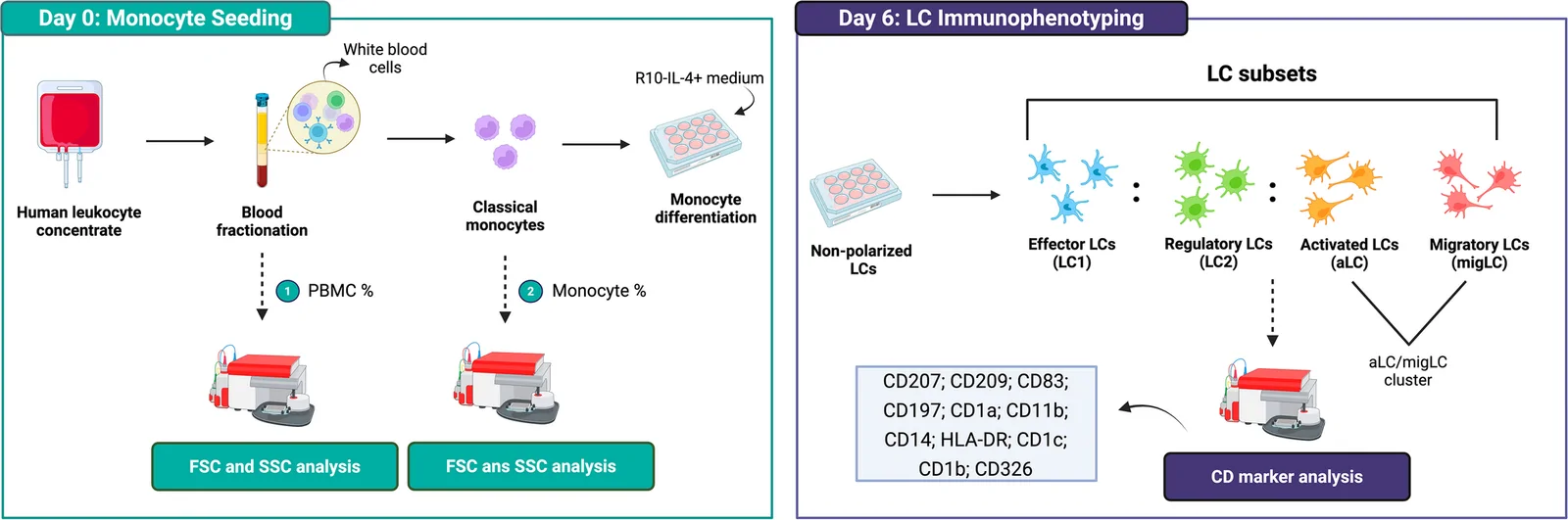
Langerhans cell (LC) differentiation method pipeline. The process includes: seeding of monocytes (day 0); complete removal of IL-4 from the culture (day 2; not visualized) by replacing 100% of the R10-IL-4 + medium with R10-IL-4-free medium; partial removal of R10-IL-4-free medium (day 4; not visualized) performed by replacing 50% of the old medium with 50% of fresh R10-IL-4-free medium; and LC immunophenotyping (day 6). Media composition, as described in Materials and methods Day 0, 2 and 4. PBMC – peripheral blood mononuclear cells; FSC – forward scatter; SSC – side scatter. Created with BioRender.com

To collect non-adherent LCs from the 12-well plates, the medium in each well was vigorously pipetted 10 times and transferred into a 50 mL tube containing 1 mL PBS (Gibco, Thermo Fisher Scientific) with 0.1% (w/v) BSA (PBS-BSA). Next, adherent LCs were washed with 1 mL of PBS, which was then added to the pooled tube. The remaining adherent cells were then detached by two sequential incubations with 0.5 mL Accutase (Invitrogen, Thermo Fisher Scientific, Waltham, MA, USA): first for 5 min at room temperature (RT) and then for 5 min at 37 °C. The eluted cells were subsequently combined with the previously pooled cell suspension and centrifuged for 5 min at 300 × g. Next, the supernatant was removed, and the cells were resuspended in PBS containing 0.1% (w/v) BSA and 0.1% (w/v) human-IgG (hIgG, Sigma-Aldrich, Burlington, MA, USA; 0.1 mL per sample to be stained). The cell suspension was then divided between Eppendorf tubes (sample = 0.1 mL/tube) to which each antibody against the selected CD markers were added, followed by 30 min incubation at 4 °C. Mouse antibodies used in the phenotyping were: Isotype controls (FITC labelled, IgG, BD Biosciences; PE labelled, IgG, and APC labelled, IgG, R&D Systems, Minneapolis, MN, USA), Anti-CD1a-FITC (Miltenyi Biotec), Anti-CD11b-APC and Anti-CD11b-Alexa Fluor 488 (BD Biosciences; BioLegend, San Diego, CA, USA), Anti-CD1b-APC (Miltenyi Biotec), Anti-CD1c-PE (Miltenyi Biotec), Anti-CD14-APC (BD Biosciences), Anti-CD83-APC (Miltenyi Biotec), Anti-CD197-PE (Miltenyi Biotec), Anti-CD207-FITC and Anti-CD207-APC (Miltenyi Biotec), Anti-CD209-PE (Miltenyi Biotec), Anti-CD326-PE and Anti-CD326-APC (Miltenyi Biotec) and Anti-HLA-DR-PE (Serotec, Kidlington, United Kingdom). Dead cells were excluded by staining with 7-aminoactinomycin D (7-AAD) (Invitrogen, Thermo Fisher Scientific). After incubation, 0.8 mL of PBS-BSA was added to each sample, followed by 5 min centrifugation at 300 × g, and removal of the supernatant. The cell pellets were then resuspended in 1% (w/v) paraformaldehyde (PFA) and analyzed by flow cytometry using an Accuri C6 PLUS flow cytometer (BD Biosciences, Franklin Lakes, NJ, USA) with compensation (triple staining) and standard settings. An overview of the LC differentiation pipeline is shown in Fig. 1.

### Staining of cells in culture chamber

#### PFA fixation

At day 6 the medium was removed from all wells of the culture chamber, and 100 µL of 4% PFA was added to each well, followed by a 20-min incubation at 4 °C. After fixation, the PFA solution was removed, and the wells were washed twice with 200 µL of PBS containing 0.1% (w/v) BSA (PBS-BSA).

#### Incubation with primary and secondary antibodies

In the next step, 100 µL of: (i) Anti-CD207 (clone 4C7, BioLegend), 10 µg/mL in PBS containing 0.1% (w/v) BSA and 0.1% (w/v) hIgG (PBA-BSA-hIgG); (ii) PBS-BSA-hIgG (control), were added to the cells. The culture chamber was then incubated for 30 min at 4 °C.

Next, the wells were washed twice with 200 µL PBS-BSA, and 200 µL rabbit anti-mouse IgG-biotin antibody (Sigma-Aldrich), 1 µg/mL in PBS-BSA-hIgG was added to each of the wells in the culture chamber, and incubated for 30 min at 4 °C.

#### SCIZYS staining and mounting

The solutions were then removed from all wells of the chamber, and the cells were washed, first with 300 µL PBS-BSA and then twice with 300 µL of SCIZYS wash buffer (Lumito AB, Lund, Sweden). Next, the cells in the wells were stained with 100 µL of SCIZYS Erbium-Streptavidin (SCIZYS Erbium-SA; Lumito AB) particles, 10 µg/mL in SCIZYS dilution buffer (Lumito AB). The chamber was incubated at RT for 60 min before the wells were washed three times with SCIZYS wash buffer for 3 min each, and air-dried. Finally, the culture chamber was removed, and the remaining slide was mounted with Prolong™ Gold Antifade Mountant with DAPI (Invitrogen, Thermo Fisher Scientific) and left overnight before scanning with the Whole-Slide Imaging Scanner, SCIZYS S1 (Lumito AB).

### SCIZYS imaging

**Fig. 2:**
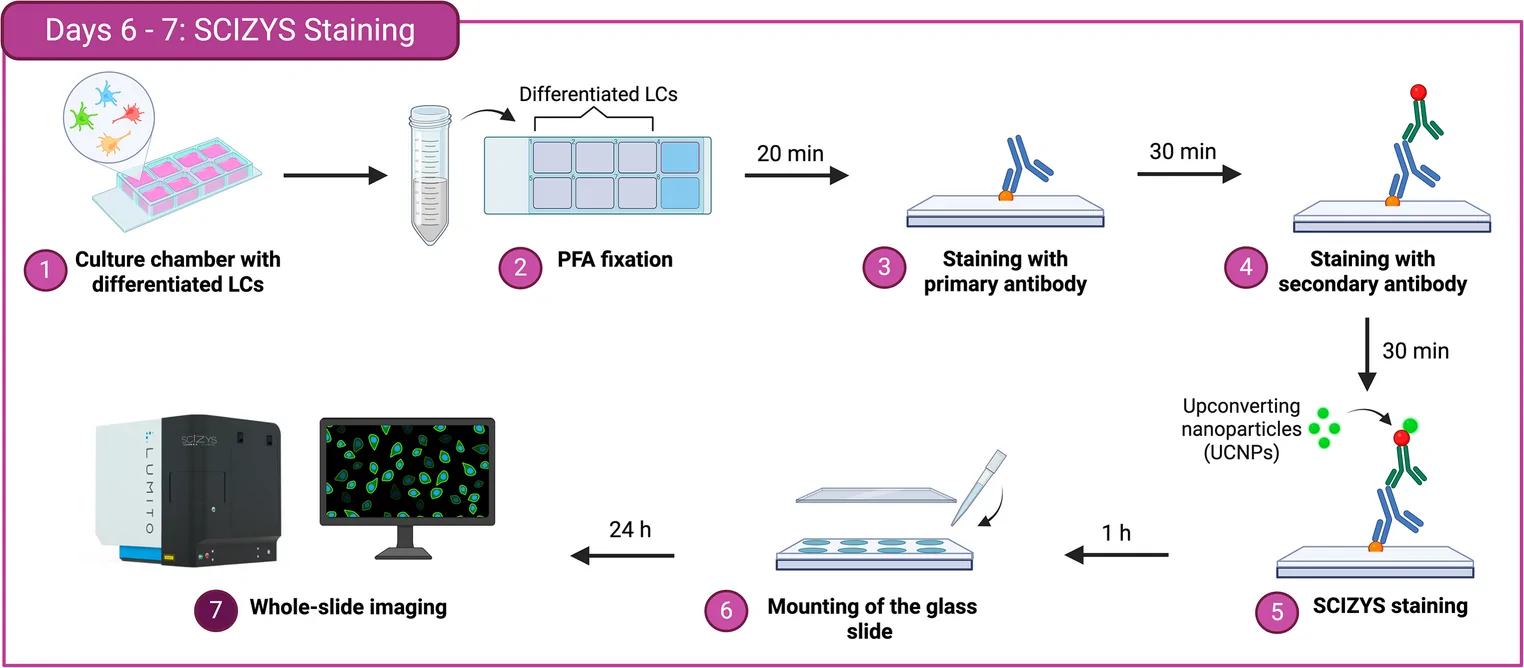
SCIZYS staining of the differentiated Langerhans cells (LCs) for whole-slide imaging using Lumito technology. The process includes paraformaldehyde fixation of the LCs; staining with primary and secondary antibodies; SCIZYS staining using upconverting nanoparticles (UCNPs); mounting of the glass slide (day 6); and whole-slide imaging using Lumito technology (day 7). PFA – paraformaldehyde. Created with BioRender.com

SCIZYS Erbium-SA is a novel label type based on photon-upconverting nanoparticles (UCNPs) [21]. These UCNPs are excited at 976 nm in the near-infrared (NIR) region, where light absorption and scattering by tissue and cells are reduced [22]. Post excitation, the SCIZYS Erbium-SA particles emit visible light with peaks at 520, 545 and 655 nm. The slide was scanned in a SCIZYS S1 system using a 20x objective (CFI Plan Apochromat Lambda D Series, Nikon Corporation, Tokyo, Japan) that combines bright-field imaging for morphological information with dark-field and upconversion luminescence microscopy to capture biomarker information. The SCIZYS S1 system can resolve individual UCNPs as diffraction-limited spots with a pixel resolution of 0.32 μm/pixel. A schematic overview of the SCIZYS staining procedure for differentiated LCs is shown in Fig. 2.

### Phagocytosing capacity of LCs and macrophages differentiated from peripheral monocytes

At day 6, 100 µL of the medium was removed from each well of the 96-well plate containing differentiated LCs and replaced with 100 µL/well of Lumogallion-labelled Alhydrogel (Lum-Alhydrogel) [23] or Alhydrogel (Brenntag Biosector, Frederikssund, Denmark), 10 µg/mL in R10. The cells were incubated overnight, eluted as described in Materials and methods Day 6, resuspended in 1% (w/v) PFA, and analyzed by flow cytometry.

As a comparative experiment, macrophages were differentiated and polarized from human peripheral monocytes [24]. Briefly, MACS isolated monocytes were differentiated to macrophages by adherence to the wells in a 96-well plate, incubation with M-CSF (Miltenyi Biotec), 20 ng/mL in serum-free medium (Gibco, Thermo Fisher Scientific) with a 100% replacement of the culture medium at day 2 and a 50% replacement at day 4. At day 6 the macrophages (M0 macrophages) were polarized into M1 and M2 macrophages and at day 7 half of the medium was removed and replaced with Lum-Alhydrogel or Alhydrogel, 10 µg/mL in SFM containing 20 ng/mL M-CSF. The cells were incubated at 37 °C in a humidified atmosphere with 5% CO_2_ for 24 h to ensure phagocytosis. The following day, the adherent cells were washed with PBS and eluted with Accutase as described in Materials and methods Day 6, resuspended in 1% (w/v) PFA and analyzed by flow cytometry.

To compare the phagocytosing capacity between the LC and the macrophage populations, the ratio between the mean fluorescence intensity (MFI) of Lum-Alhydrogel and Alhydrogel from each staining was calculated. Statistical analyses were performed using parametric one-way Welch ANOVA and Brown-Forsythe tests followed by Games-Howell post-hoc test to assess differences between groups. Due to the small sample sizes, results were additionally confirmed using the non-parametric Kruskal-Wallis test. A *p*-value < 0.05 was considered statistically significant.

### Software and statistical analysis

The flow cytometry data were analyzed using BD Accuri C6 software version 1.0.34.1 (BD Biosciences). QuPath version 0.6.0 was used for image assessment and generating overlays [25]. Data are presented as mean ± standard deviation (SD), based on 13 independent differentiation experiments unless otherwise noted in figure legends. Data analysis and visualization were performed using GraphPad Prism, version 10.1.0 (GraphPad Inc.; San Diego, CA, USA).

## Results

### Identification of monocyte-derived Langerhans cell subsets via CD marker expression using flow cytometry

First, to characterize LC subsets derived in vitro from human peripheral blood monocytes, the expression of specific CD markers was evaluated by flow cytometry. To identify LC subsets, the CD panel described by Liu et al., was used [12]. Table 1 shows the differential expression levels of CD markers that define distinct LC subsets reported by Liu et al. [12], ranging from low to high, including intermediate expression levels such as low-medium and medium.

**Table 1:** Expression of CD markers by Langerhans cell (LC) subsets as reported by Liu et al. [12]

LC subsets	CD marker expression levels						
	CD207	CD1a	CD1b	CD1c	CD83	CD197	HLA-DR
LC1	high	high	low	low	low	low	low-medium
LC2	low-medium	medium	low-medium	high	low	low	low-medium
aLC	medium	low	low-medium	low	high	low	high
migLC	medium	low	low-medium	low	high	high	high

**Fig. 3:**
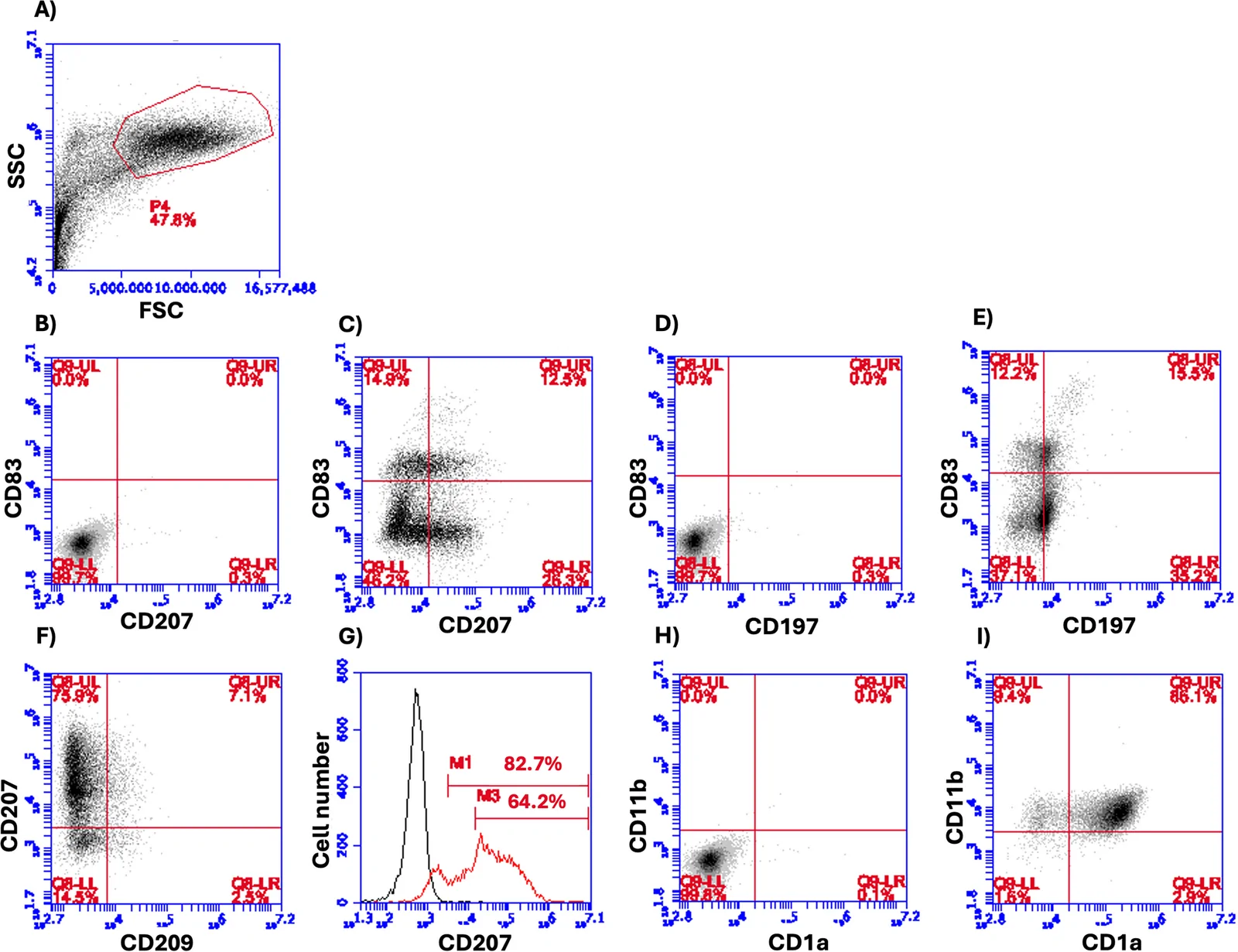
Flow cytometry analysis of CD marker expression used to distinguish Langerhans cell (LC) subsets. **A** Scatter plot (FSC vs. SSC) showing total LC population as gate P4; **B** Isotype control that applies to the CD207 and CD83 expression; **C** CD207 and CD83 expression; **D** Isotype control that applies to the CD197 and CD83 expression; **E** CD197 and CD83 expression; **F** CD209 and CD207 expression; **G** Histogram showing the CD207 expression in red and isotype control in black; **H** Isotype control that applies to the CD1a and CD11b expression; **I** CD1a and CD11b expression. Results from one representative donor out of thirteen is shown in the figure. FSC – forward scatter; SSC – side scatter; M1 – LCs characterized by low-medium to high CD207 expression; M3 – LCs characterized by medium to high CD207 expression

After differentiating human peripheral monocytes into LCs, viable cells were identified for each donor using forward scatter (FSC) and side scatter (SSC) parameters and gated as exemplified in Fig. 3A as gate P4. Next, the joint aLC/migLC cluster was identified for each donor based on the expression of CD83, CD207, and CD197 markers [12] as shown in Fig. 3C and E. The joint aLC/migLC cluster was characterized as double-positive cells expressing either CD207^medium/high^, CD83^high^ or CD197 ^low/high^, CD83^high^, which in the example shown in the figure corresponded to 12.5% (Fig. 3C) and 15.5% of double-positive cells (Fig. 3E), respectively. The assumed margin of error was set at ± 10% between both staining types. Since the same CD83 antibody was used in both stainings, the overall CD83^+^ expression was assessed and found to be comparable with values of 27.4% (Fig. 3C) and 27.7% (Fig. 3E), which indicates good agreement between the results. However, in the further calculations, only the CD197^+^CD83^+^ values were used to define the aLC/migLC cluster, corresponding to 15.5% in the example shown.

Next, a CD207⁺ cell population (CD207^+^ _Tot_) was identified, comprising approximately 83% of the cells (Fig. 3F), including a double-positive CD207^+^CD209^low^ population accounting for 7.1%. In parallel, a double-negative CD207CD209 population, represented 14.5% of all cells (Fig. 3F) in the P4 gate (Fig. 3A). Together, these distributions indicate a donor with a relatively high proportion of the generated LCs.

Since all LCs expressed CD207 at levels ranging from low-medium to high, a histogram of CD207 expression versus cell number was used to define the M1 and M3 gates (Fig. 3G). The M1 gate, representing all CD207⁺ cells, encompassed 82.7%, which closely matched the 83% CD207⁺_Tot_ population based on Fig. 3F. The M3 gate was set according to the CD207⁺ threshold defined in Fig. 3C (x-axis > 10^4^), capturing medium-to-high CD207⁺ populations and accounting for 64.2%.

The LC1 and LC2 subsets were calculated using Eqs. 1 and 2, respectively. In Fig. 3G, M1 = 82.7%, and M3 = 64.2%, and in Fig. 3E, aLC/migLC = 15.5%, therefore LC1 = 48.7%, while LC2 = 18.5%.

1 $$\:M3\:\left({CD207}^{medium-high}\right)=LC1+aLC/migLC$$

2 $$\:M1\:\left({CD207}_{Tot}^{+}\right)=LC1+LC2+aLC/migLC$$

3 $$\:M1\:\left({CD207}_{Tot}^{+}\right)={LC}_{Tot}$$

Next, using Eq. 3, the total percentage of LCs (CD207^+^ _Tot_) was compared to the overall percentage of CD1a⁺ cells (LC_Tot_) (Fig. 3I). The assumed margin of error was set at ± 10%. The calculated CD207^+^ _Tot_ was 83%, while the CD1a⁺ population constituted 89% of all cells, indicating strong alignment between the two measurements (86% ± 3%).

The differentiated LCs lacked CD14 expression (Online Resource; Fig. S1B), and roughly 96% of the cells expressed CD11b at medium to high levels (Fig. 3I). Additionally, all generated LCs consistently expressed HLA-DR at low-medium to high levels across donors (Online Resource; Fig. S1B), while also expressing CD1b and CD1c (Online Resource; Fig. S1E).

It is also important to note that atypical LC characteristics were obtained after in vitro differentiation of peripheral blood monocytes from three out of the 16 donors. The cells obtained from these three donors displayed 51–66% of a double-negative CD207CD209 phenotype (Online Resource; Fig. S2B). At the same time, the CD207CD209 double-negative cells were CD1a^+^CD11b^+^ (Online Resource; Fig. S2E). Since this unexpected cell population did not represent a canonical LC phenotype, these donors were excluded from further analysis.

### Visualization of monocyte-derived Langerhans cells via SCIZYS whole-slide imaging technology

To visualize LCs differentiated in vitro from monocytes, the cells were stained for CD207 expression using the SCIZYS technology that, in contrast to conventional fluorophores, utilizes particles that emit light with shorter wavelengths, a so-called anti-Stokes shift [26]. The combination of NIR excitation and anti-Stokes emission eliminates background signals, such as autofluorescence, that are issues in traditional fluorescence microscopy [22]. The technology combines the use of UCNPs with bright-field microscopy and AI-driven image analysis.

**Fig. 4:**
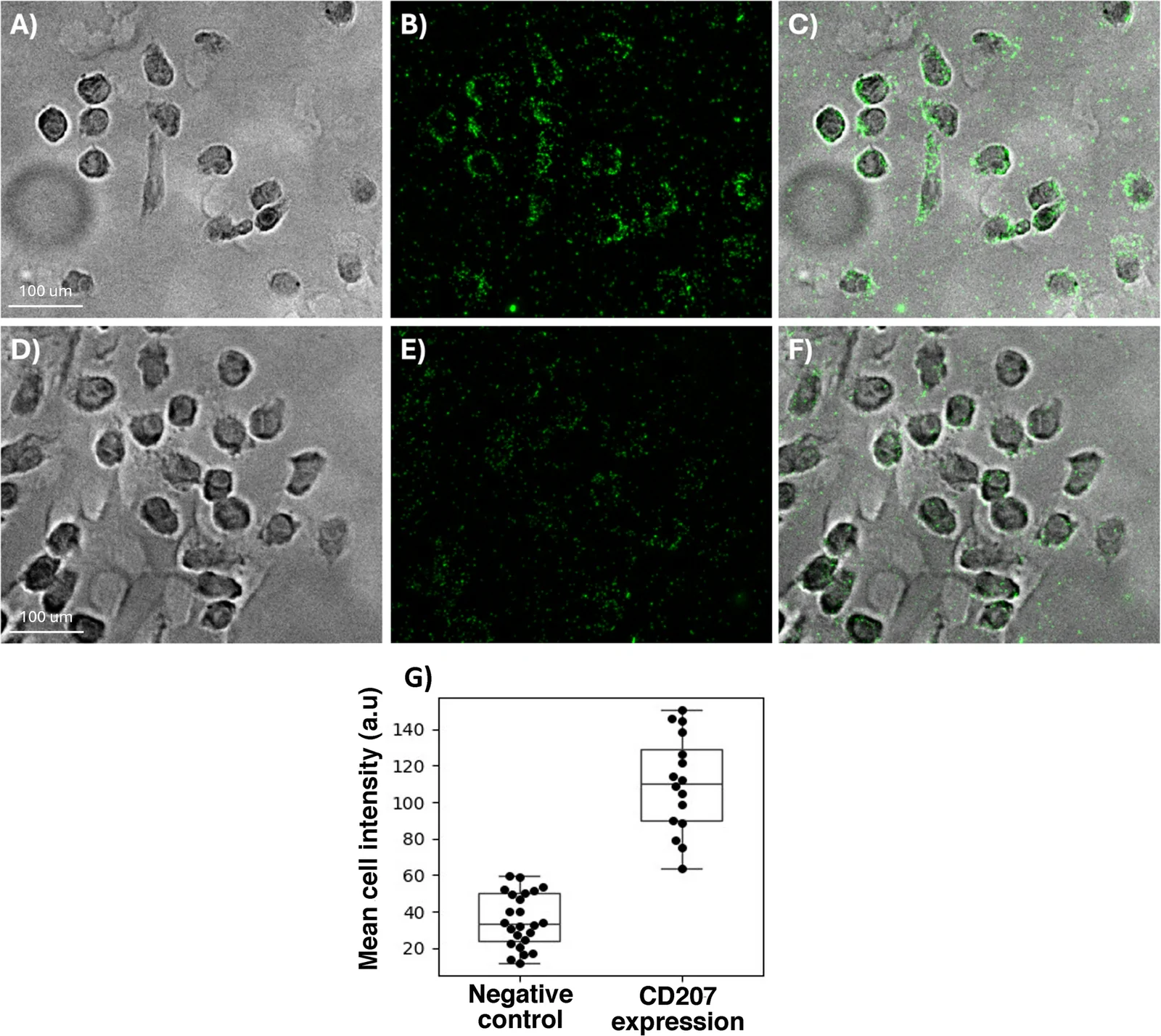
Visualization of monocyte-derived Langerhans cells (LCs) by CD207 expression using SCIZYS whole-slide imaging technology. **A** Bright-field image of LCs stained against CD207; **B** Upconversion luminescence image of LCs stained against CD207; **C** Composite image of LCs stained against CD207; **D** Bright-field image of LCs negative control; **E** Upconversion luminescence image of LCs negative control; **F** Composite image of LCs negative control; **G** Quantified luminescence from LCs stained with anti-CD207 (**4B**) and LC negative control (**4E**). Box and its central line depict the median and interquartile range. For the negative control, LCs were processed without the primary anti-CD207 antibody during the staining procedure. Scale bars are 100 μm

SCIZYS whole-slide imaging technology enabled clear and detailed visualization of the CD207 expression by the monocyte-derived LCs (Fig. 4). The composite image of LCs stained against CD207 shows both low- and high-expressing cells, consistent with the results obtained by flow cytometry.

### Quantification of distinct monocyte-derived Langerhans cell subsets and donor variability

The LC1, LC2 and aLC/migLC values for each donor were then used to quantify the mean percentage of each LC subset. The exact percentages of distinct LC subsets across different donors along with total LC population (LC_Tot_) and mean values are shown in Table 2.

**Table 2:** Variation in the percentages of distinct Langerhans cell (LC) subsets and total LC population (LC_Tot_) across different donors along with mean values for all donors (*n* = 13)

Donor number	LC subset percentage (%)			CD207^+^ _Tot_ (%)	LC_Tot_ (%)
	LC1	LC2	aLC/migLC		
1	47.9	11.7	20.1	79.7	89.7
2	55.9	10.3	14.7	80.9	91.7
3	58.0	13.1	19.0	90.1	97.0
4	32.1	20.5	16.6	69.2	85.6
5	62.6	12.9	19.6	95.1	98.3
6	67.1	19.7	10.0	96.8	98.4
7	31.0	15.4	9.4	55.8	88.1
8	62.1	18.6	16.5	97.2	98.7
9	33.7	23.2	19.0	75.9	74.7
10	49.3	14.2	15.9	79.4	91.0
11	48.7	18.5	15.5	82.7	89.0
12	40.4	10.2	18.8	69.4	84.4
13	44.5	19.6	5.2	69.3	84.4
Mean ± SD	**48.7 ** **± 12.1**	**16.0** ** ± 4.3**	**15.4 ** **± 4.6**	**80.1 ** **± 12.5**	**90.1 ** **± 7.0**

The mean percentages of steady-state LC subsets were ~ 49% for LC1 and 16% for LC2. In contrast, the inflammation-associated aLC/migLC cluster had a mean percentage of ~ 15%. Overall, the average total yield of LCs obtained through in vitro differentiation of human peripheral monocytes was ~ 80%.

**Fig. 5:**
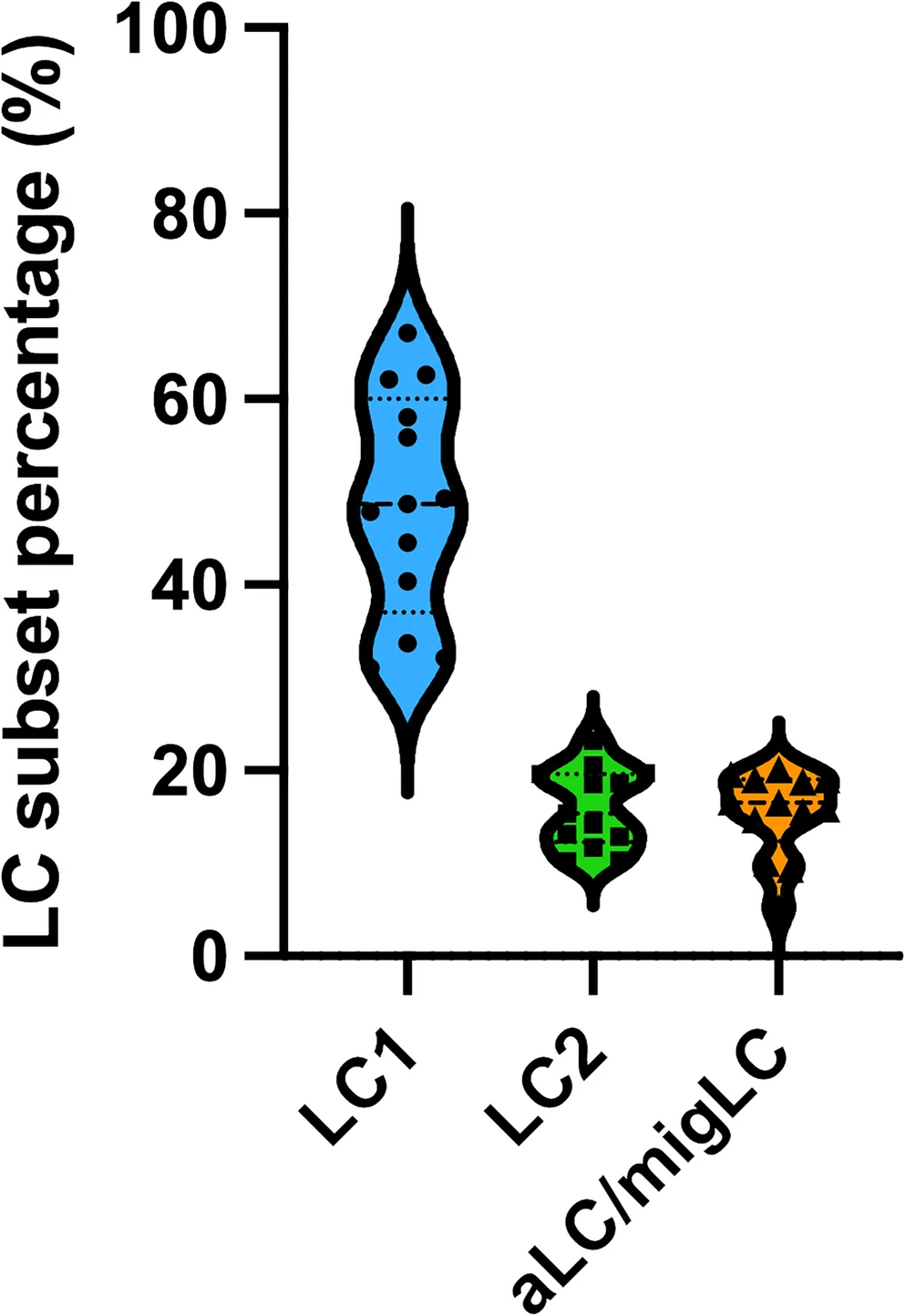
Distribution differences of Langerhans cell (LC) subsets across various donors. Violin plot depicting the distribution shape of the data (*n* = 13)

Next, to evaluate how LC distributions varied among donors, we analyzed differences across individual samples. The experimental system revealed inter-donor variability in LC subset distribution, highlighting individual donor-dependent differences. The distribution differences of LC subsets across various donors are shown in Fig. 5.

### Expression of CD326 (EpCAM) by the monocyte-derived Langerhans cells

Next, we investigated the expression of Epithelial Cell Adhesion Molecule (EpCAM; CD326) on CD207^+^CD11b^+^ monocyte-derived LCs using both triple staining for all three markers and double staining for EpCAM and CD207 (Online Resource; Fig. S3). The results showed that the triple-staining pattern correlated well with the double staining; however, only approximately 4–6% of the LCs were weakly positive for EpCAM (Online Resource; Fig. S3D).

### Comparison of the phagocytotic capacity between different classes of macrophages and monocyte-derived Langerhans cells

Given the dual nature of LCs we investigated the phagocytic capacity of the in vitro-derived LCs using fluorescent labelled aluminum-based adjuvants as traceable particles [23]. The phagocytosing capacity of the LCs was compared with different polarizations of in vitro monocyte-derived macrophages.

**Fig. 6:**
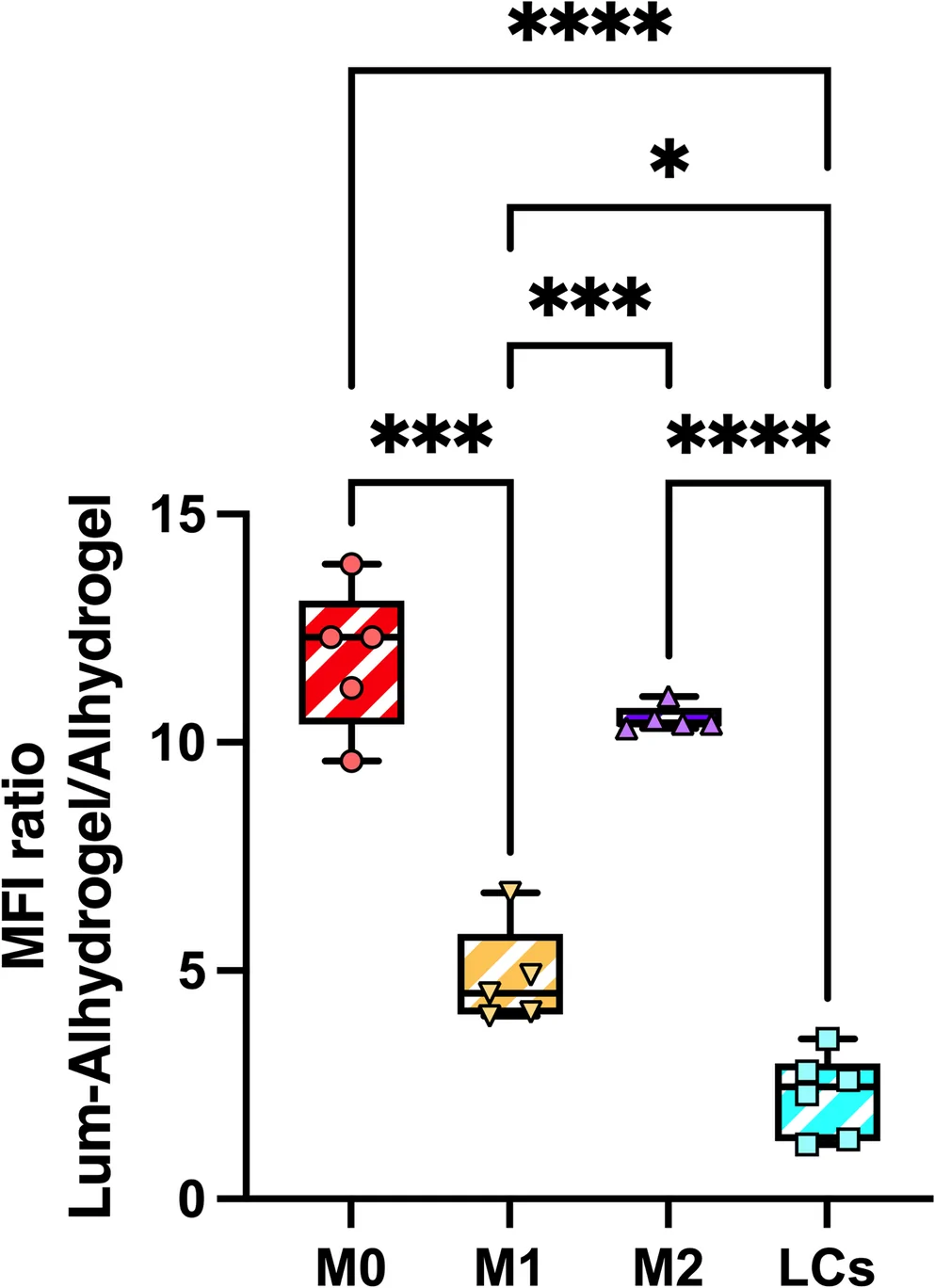
Comparison of phagocytic capacity between different macrophage polarizations (M0, M1, M2) and monocyte-derived Langerhans cells (LCs). Data are presented as box-and-whisker plots showing median and interquartile range, with whiskers indicating the range. Phagocytic capacity is expressed as the MFI ratio between Lum-Alhydrogel and Alhydrogel where *n* = 5 (M0, M1, M2) and *n* = 6 (LCs). A *p*-value of < 0.05 (*p* < 0.05) was considered statistically significant (one-way Welch ANOVA and Brown-Forsythe tests). * *p* ≤ 0.05 *** *p* ≤ 0.001 **** *p* ≤ 0.0001. Lum-Alhydrogel – Lumogallion-labelled Alhydrogel; MFI – mean fluorescence intensity

The data presented in Fig. 6 indicate that relative to the various macrophage polarization states, monocyte-derived LCs displayed the lowest phagocytic capacity in response to aluminum-based adjuvants, in contrast to M0, M1, and M2 macrophages. Within the total LC population, phagocytic activity was predominantly attributable to the aLC/migLC and LC2 subsets, whereas the LC1 subset remained negative or nearly devoid of phagocytic function. Nevertheless, evaluation across a larger donor cohort will be required to establish statistically robust conclusions.

## Discussion

Langerhans cells (LCs) were first described over a century ago [27] and are epidermal APCs involved in both immune activation and tolerance. However, despite extensive study their biology remains incompletely understood.

Human LCs are present at low abundance in the epidermis, and their isolation is labor-intensive and requires large amounts of skin tissue; as a result, obtaining sufficient cell numbers for more comprehensive studies remains a major obstacle [1]. 

In vivo, epidermal LCs arise from embryonic myeloid precursors, forming a long-lived, self-renewing population within the skin [28]. However, several studies have shown that bone marrow-derived myeloid cells can also replenish the LC pool under inflammatory or even steady-state conditions [3, 7, 29]. The *de novo* recruitment of monocytes during severe immune injury remains poorly understood [30]. Several studies have proposed a two-wave model, in which classical monocytes first transiently replenish the LC niche, followed by a second wave of uncharacterized myeloid precursors that give rise to a long-lived LC population [7, 9]. The situation is further complicated by contradictory findings on whether donor monocytes can fully reproduce the properties of steady-state eLCs. Some studies indicate that donor monocytes serve as precursors that repopulate the depleted epidermal compartment, giving rise to the long-term LC population comparable to that of steady-state eLCs [3]. Despite considerable research, the mechanisms underlying monocyte-driven epidermal LC repopulation, and whether it occurs transiently or persistently, remain poorly understood [9].

Differentiation of LCs from peripheral blood monocytes was done according to a modification of the protocol described by Otsuka et al. [31]. Several modifications were exploited and introduced as a part of this study. The modifications involved procedural adjustments on days 2 and 4, including the complete replacement of the IL-4-containing culture medium on day 2 with fresh IL-4-free medium, instead of the partial medium exchange described in the original protocol. Additionally, in our method, non-adherent cells present in the IL-4-containing medium on day 2 were retained by centrifugation and reintroduced into the culture. The retention of non-adherent cells was also introduced on day 4, where, although the IL-4-free medium was exchanged partially, similar to the original protocol, great care was taken to avoid the removal of non-adherent cells from the culture. The implementation of these modifications resulted in a higher overall yield of LCs than in the original method [31].

In this study, LCs were differentiated from 16 donors, of which 13 were retained for further analysis. Retention criteria required that at least 50% of the differentiated population consists of CD207⁺ cells, and that the double-negative CD207CD209 population comprises less than 30% of all differentiated cells. For this reason, three of the 16 donors who did not meet the established criteria were excluded from further analysis, representing less than 20% of the total donor pool investigated in this study. Interestingly, donors that gave rise to a high proportion of double-negative cells could often be identified as early as on day 4 by the absence or low number of non-adherent cells in the culture. This observation not only enables early decisions on whether to continue with a given donor, saving time and resources, but also underscores the importance of retaining non-adherent cells in the culture when changing the culture medium on both days 2 and 4.

Recent studies have revealed the presence of phenotypically and functionally distinct LC subsets in human skin, shedding light on their intra-populational heterogeneity [12, 13]. Utilizing CD marker profiling, LCs have been classified into four distinct subsets: LC1 (CD207^high^, CD1a^high^) and LC2 (CD207^+^, CD1a^low^, CD1b^+^, CD1c^+^), present at steady state, along with aLC (CD207^+^, CD83^+^, CCR7^−^) and migLC (CD207^+^, CCR7^+^) found during skin inflammation [12], and summarized in Table 1. All these subsets have been confirmed to be present both in normal human skin and in LCs derived from CD34⁺ hematopoietic stem cells (HSC) isolated from cord blood [12, 14]. LC1 and LC2 represent steady-state subsets that originate from preLCs along two distinct developmental branches [12]. However, LC1 subset exhibits greater self-renewal capacity and enhanced antigen uptake, and secretes cytokines associated with innate immune responses. In contrast, LC2 subset displays high expression of genes involved in regulating immune effectors and produces immunomodulatory factors characteristic of adaptive immunity [14]. Although LC1 subset is more resistant against activation under inflammatory conditions than LC2, both subsets can give rise to aLC, while only aLC can give rise to migLC subset [12]. Following the initial CD-marker characterization and selection, LCs from 13 donors were analyzed for the relative proportions of distinct LC subsets within the obtained cell populations (Table 2; Fig. 5). Due to the low overall abundance of aLC and migLC subsets within the total LC population, these cells were analyzed together and, therefore, were referred to as aLC/migLC cluster. Analysis revealed that steady-state LC1 and LC2 subsets comprised between 31–67% and 10–23% of the differentiated cell populations, respectively. Concurrently, the combined inflammatory aLC/migLC cluster comprised 5–20% of the cells across donors (Table 2), placing its abundance closer to the proportions of the aLC/migLC cluster reported in human skin (5–8%) than HSC-derived LCs (~ 33%) reported by Liu et al. [12]. The results of LC1 subset (31–67%) corresponded well with the proportions obtained from both HSC-LCs and skin-derived LCs, 37–62%, whereas the content of LC2 subset, 10–23%, was lower compared to what has been reported from HSC-LCs and skin-derived LCs (around 30%).

In the study by Liu et al. [12], the higher proportion of aLC/migLC cluster in HSC-derived cells was attributed to in vitro conditions potentially promoting LC activation and migration, in contrast to normal human skin, which may also explain the results observed in our study. However, it is worth noting that in the study by Liu et al. [12], the reference samples of normal skin were obtained from human foreskin, which, due to its anatomical location and the young age of the donors, likely exhibited a lower baseline activation [32], contributing to a reduced proportion of aLC/migLC cluster. Nevertheless, the fact that most of the LCs generated in our study exhibit a steady-state phenotype is advantageous, as it allows them to be subsequently exposed to various stimuli to induce activation, making them well-suited for studying LC activation and functional responses under controlled conditions.

It should also be stated that the low variability between the donors within the LC2 and aLC/migLC subsets suggests that these populations are relatively stable across donors. Conversely, the LC1 subset displayed greater variation, indicating higher donor-dependent heterogeneity (Table 2; Fig. 5). This indicates that donor-specific differences exist within the tested subject pool, and some level of variability in the proportions of LC subsets differentiated from human peripheral monocytes across independent experiments is to be expected.

Epithelial Cell Adhesion Molecule (EpCAM; CD326) is a transmembrane glycoprotein reported to be selectively expressed on the surface of mouse epidermal LCs, where it mediates intercellular adhesion [33]. However, although the expression of EpCAM has been reported by LCs in murine models [9], previous studies have also shown that human LCs do not express EpCAM, but rather its closely related homolog, TROP2 [34]. In this study, we observed that among monocyte-derived LCs, only a small fraction of CD207^+^CD11b^+^ cells were weakly EpCAM^+^ (around 4–6%), suggesting that our observations may reflect a species-specific difference, consistent with previous reports [34]. Most investigations into LC biology, including those distinguishing long- and short-term LC populations [9], have been conducted in murine models [35]. This makes the proposed explanation plausible and underlies the inherent challenges in using animal models to predict human LC biology. Taken together, such conflicting evidence from the existing literature highlights that LC biology is highly complex and remains incompletely understood.

Like macrophages derived from monocytes, LCs can migrate to draining lymph nodes and present antigens to antigen-specific T cells, which is consistent with the adoption of a macrophage and dendritic cell-like features by the LCs upon activation and differentiation [2]. Consequently, LCs have been described as possessing a dual identity, functioning as “macrophages in dendritic cell clothing” [36]. Phagocytosis is a fundamental mechanism of macrophages and immature dendritic cells (DCs) to internalize and eliminate foreign pathogens [37], which, upon inflammatory conditions enables these immune cells to act as APCs. Beyond its role in host defence, phagocytosis is indispensable for the removal of apoptotic cells and cellular debris in both homeostatic and inflammatory contexts, thereby serving as the body’s housekeeping system that preserves tissue integrity and immune balance [38]. Comparative analysis of the phagocytic capacity of aluminum-based adjuvants revealed that, unlike in vitro differentiated and polarized M0 macrophages and alternatively activated M2 macrophages, which displayed high phagocytic activity, LCs derived through in vitro differentiation exhibited low phagocytic capacity (Fig. 6). The phagocytic capacity of the total LC population was the lowest among all cell types examined, and results indicating the phagocytosing capacity of the LCs were mainly mediated by the aLC/migLC and LC2 subsets. This suggests that LCs might be more oriented toward immune-regulatory functions rather than general “housekeeping” or debris clearance. This aligns well with the notion that upon maturation, LCs adopt more dendritic cell-like functions, resulting in reduced phagocytic activity in favour of enhanced antigen presentation through the MHC pathway [39]. Another possible explanation is the inherent reduction in phagocytic capacity upon LC maturation in in vitro culture, as reported in an earlier study, although the absence of more recent studies suggests that its conclusions may not fully capture the current state of knowledge [40]. Additional explanations include suboptimal particle size of the Alhydrogel^®^-adjuvants (1–5 μm), which exceeds the maximal phagocytotic limit of < 0.5 μm by LCs [38], along with possible immune tolerance to aluminum-based adjuvants resulting from prior donor exposure to similar particles through routine vaccination programs [41].

Interestingly, further analysis of the double-negative CD207CD209 population from the three excluded donors revealed these cells were clearly CD1a^+^CD11b^+^ (Online Resource; Fig. S2E). We initially hypothesized that these cells were monocyte-derived DCs (moDCs); however, literature evidence contradicted this assumption. While moDCs are CD207^negative^, unlike the unknown cells, they express high levels of CD209. This along with evidence that only approximately 40% of moDCs express CD1a, eliminates these cells as potential candidates [42]. We also considered inflammatory dendritic epidermal cells (IDECs) as a possible identity for this population, given their reported CD207^negative^CD1a^+^ phenotype. However, IDECs are known to express CD209 rather than be CD209^negative^, excluding this possibility [31, 43–45]. Another option was that these cells were associated with indeterminate dendritic cell histocytosis (IDCH), a rare and poorly characterized disorder, due to their CD207^negative^CD1a⁺ phenotype [46]. However, the literature does not clearly define their CD209 expression profile, leaving it uncertain. The most plausible explanation seems to be the dermal CD1a^+^ DC population, which has been shown to exhibit CD207^negative^, CD1a^+^, and CD209^negative^ phenotype in human skin [47]. However, as the atypical population reported in our study was found in fewer than 20% of the donors analyzed, additional studies will be required to verify this possibility. 

In vivo, epidermal LCs maintain the local immune network through reciprocal crosstalk with keratinocytes. Interleukin 34 (IL-34) is a keratinocyte-derived factor that is crucial for the terminal differentiation of LC precursors during embryogenesis and for maintaining their self-renewal capacity throughout adulthood [48, 49]. Interestingly, IL-34 does not seem to be required for the inflammation-induced monocyte influx into the epidermis, and their subsequent differentiation into LCs. However, it has been shown to be critical for LC survival after inflammation once homeostasis is restored [50]. Given that in this study, LCs were generated in vitro under monoculture conditions, the phenotypic traits described here apply solely to cells derived in this context. Consequently, the cells generated in vitro under this simplified system may lack certain LC characteristics that emerge from the interactions with keratinocyte-derived molecules. Therefore, future studies should employ co-culture systems incorporating keratinocytes to promote the acquisition of in vivo-like phenotypic traits associated with LC maintenance and survival under steady-state conditions.

Given the functional duality of LCs in mediating both tolerogenic and immunogenic responses [10, 11], they have also been implicated in a range of cutaneous pathologies, including skin cancers, i.e., melanoma, and autoimmune disorders, i.e., psoriasis. However, findings regarding the role of LCs in these conditions remain inconclusive, with evidence supporting both immunogenic and immunosuppressive functions [6, 51–53]. Interestingly, recent studies suggested that heterogeneity of LC subsets may account for the inconsistent findings concerning their involvement in the progression of these diseases. For example, Dong et al. [54]., demonstrated that depletion of resident LCs (rLCs) in murine models, which phenotypically resembled the LC1 subset, accelerated the recruitment and accumulation of monocyte-derived LCs (moLCs) within melanoma lesions, thereby promoting disease progression and resistance to immunotherapy. Subsequently, Liu et al. [12]., investigated the presence of LC subsets in psoriatic skin from human donors revealing a significant increase in the numbers of LC2 and aLC/migLC subsets, but not LC1 subset, in psoriatic lesions compared with healthy skin. These findings suggest that the chronic inflammatory environment associated with systemic immune dysregulation in psoriasis [55, 56] drives LCs toward more regulatory or differentiated phenotypes. However, the precise immune-regulatory functions of these subsets in psoriasis and melanoma remain to be elucidated. In vitro modulated skin containing LCs is therefore required to further investigate their functional importance in these skin conditions. To conduct this type of study, easy access to human LCs is necessary.

Langerin (CD207) is a C-type lectin receptor involved in antigen capture and presentation during adaptive immune responses. It is highly expressed by LCs in the skin, predominantly on their surface, but also internally within Birbeck granules [57, 58] and is regarded as a marker for LCs. In this study, the expression of CD207 and CD1a was used as a key indication of the presence of the LC phenotype. The various LC subsets were identified by flow cytometry (Fig. 3; Table 2). These observations, coupled with the high levels of CD1a expression (Fig. 3; Table 2), further support the notion that the cells generated through in vitro differentiation of peripheral monocytes in this study are indeed LCs. This work describes how different LC subsets can be generated from human peripheral blood monocytes.

## Conclusions

In this study, in vitro differentiation of human peripheral blood monocytes into LCs resulted in the generation of three distinct subsets: LC1, LC2, and a joint aLC/migLC cluster. Among these, the steady-state LC1 subset was present at a higher proportion than the LC2 and inflammation-associated aLC/migLC subsets. The experimental system also revealed inter-donor variability in LC subset distribution, highlighting individual donor-dependent differences. Collectively, these findings demonstrate that in vitro differentiation of LCs from peripheral monocytes can be used to generate phenotypically distinct LC subsets. Consequently, this system offers a valuable platform for advancing our understanding of human LC biology and heterogeneity and lays the groundwork for developing more advanced in vitro models to facilitate investigations into the role of LCs in the normal skin and in the pathogenesis of various skin diseases.

## Supplementary Information

Below is the link to the electronic supplementary material.

Supplementary Material 1

## Data Availability

The raw data that support the findings of this study are available upon request from the corresponding authors.
