# Supplementary material for: In vitro differentiation and phenotypic profiling of Langerhans cell subsets derived from human peripheral blood monocytes
Source: Immunol Res. 2026 Sep 7;74(1):103. doi: 10.1007/s12026-026-09825-8 (PMC13550069; doi:10.1007/s12026-026-09825-8)
Supplement: Supplementary file 1 [file 12026_2026_9825_MOESM1_ESM.pdf]

Supplementary Information

In vitro differentiation and phenotypic profiling of Langerhans cell subsets derived from human peripheral blood monocytes

Sylwia Hasterok[1,2,*], Klas Berggren[3], Claes af Klinteberg[3], Håkan Eriksson[1,2,*], Lars Ohlsson[1,2], & Anna Gustafsson[1,2]

[1] Department of Biomedical Science, Faculty of Health and Society, Malmö University, Malmö, Sweden

[2] Biofilms – Research Center for Biointerfaces, Malmö University, Malmö, Sweden

[3] Lumito AB, Lund, Sweden

[*] Correspondence: sylwia.hasterok@mau.se; hakan.eriksson@mau.se. Tel.: (+46) 40-665 88 22; (+46) 40-665 79 25

S1. Expression profile of additional CD markers for identification of monocyte-derived Langerhans cells by flow cytometry

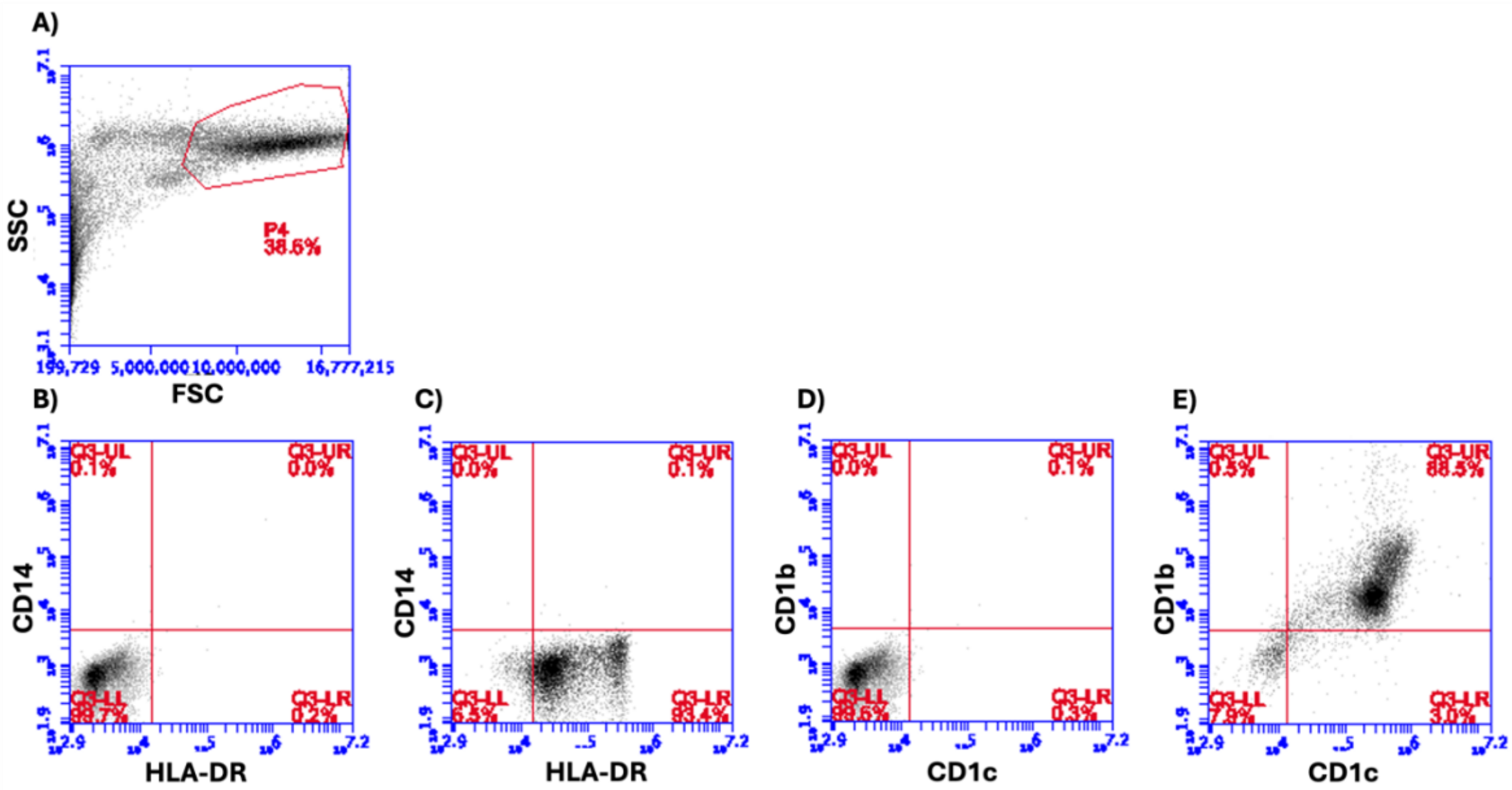

Fig. S1 Flow cytometry analysis of HLA-DR, CD14, CD1c and CD1b expression by *in vitro* differentiated LCs derived from human peripheral monocytes. **A)** Scatter plot (FSC vs SSC) showing the P4 gate identifying the cell population analyzed; **B)** Isotype control that applies to the HLA-DR and CD14 expression; **C)** HLA-DR and CD14 expression; **D)** Isotype control that applies to the CD1c and CD1b expression; **E)** CD1c and CD1b expression. Results from one representative donor are shown in the figure. FSC – forward scatter; SSC – side scatter

S2. Expression profile of CD markers by atypical cell population of undefined identity

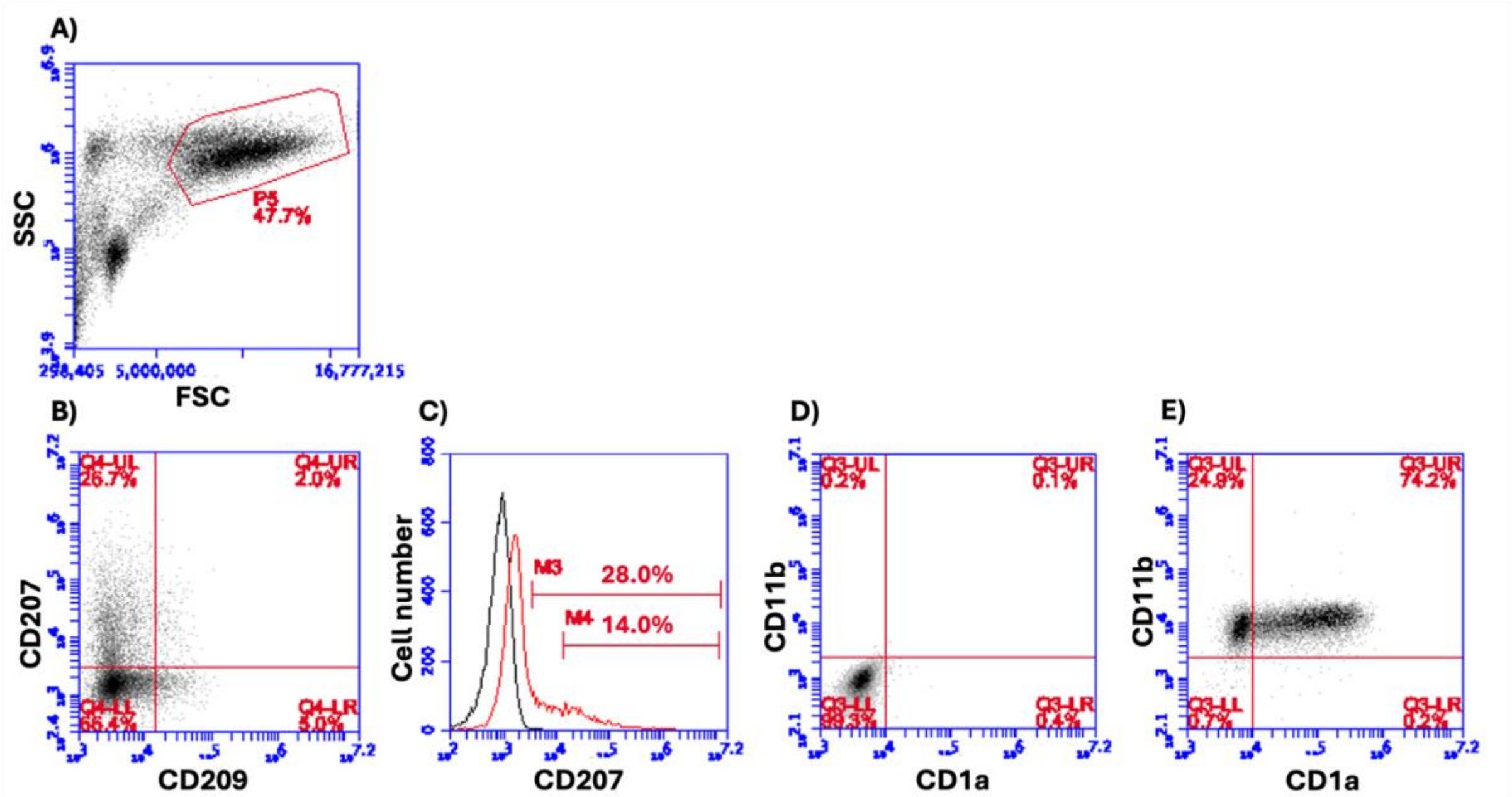

Fig. S2 Flow cytometry analysis of CD marker expression used to distinguish the atypical cell population detected in three out of sixteen donors. **A)** Scatter plot (FSC vs SSC) showing the P5 gate identifying the cell population analyzed; **B)** CD209 and CD207 expression; **C)** Histogram showing the CD207 expression in red and isotype control in black; **D)** Isotype control that applies to the CD1a and CD11b expression; **E)** CD1a and CD11b expression. Results from one representative donor are shown in the figure. FSC – forward scatter; SSC – side scatter; M3 – cells characterized by low-medium to high CD207 expression; M4 – cells characterized by medium to high CD207 expression

S3. Expression of EpCAM (CD326) by the monocyte-derived LCs

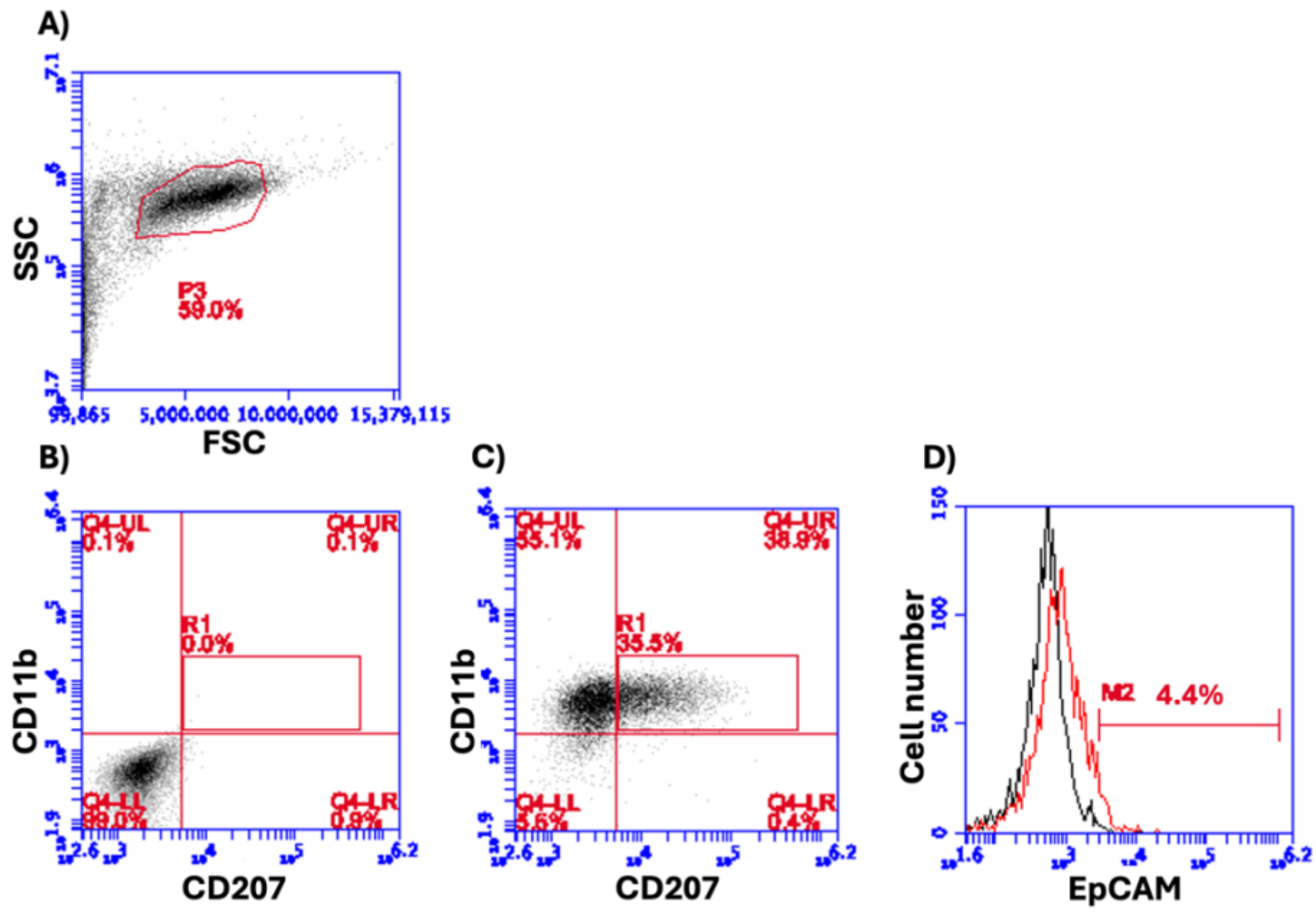

Fig. S3 Flow cytometry analysis of EpCAM expression profile by the double-positive CD207CD11b monocyte-derived LCs. **A)** Scatter plot (FSC vs SSC) showing the P3 gate identifying the cell population analyzed; **B)** Isotype control that applies to the CD207 and CD11b expression; **C)** CD207 and CD11b dot plot showing the R1 gate used to identify the EpCAM expression by the CD207CD11b double-positive population; **D)** Histogram showing the EpCAM expression by the CD207CD11b double-positive cells.

Isotype control in black and cells stained against the EpCAM in red. Results from one representative donor are shown in the figure. FSC – forward scatter; SSC – side scatter; M2 – cells characterized by the EpCAM expression
